# Decreased long noncoding RNA SPRY4-IT1 contributing to gastric cancer cell metastasis partly via affecting epithelial–mesenchymal transition

**DOI:** 10.1186/s12967-015-0595-9

**Published:** 2015-08-04

**Authors:** Min Xie, Feng-qi Nie, Ming Sun, Rui Xia, Yan-wen Liu, Peng Zhou, Wei De, Xiang-hua Liu

**Affiliations:** Department of Biochemistry and Molecular Biology, Nanjing Medical University, Nanjing, 210029 Jiangsu People’s Republic of China; Department of Oncology, First Affiliated Hospital, Nanjing Medical University, Nanjing, People’s Republic of China; The First Clinical Medical College of Nanjing Medical University, Nanjing, People’s Republic of China

**Keywords:** Long noncoding RNA, SPRY4-IT1, Gastric cancer, Proliferation and invasion, EMT

## Abstract

**Background:**

Long noncoding RNAs (lncRNAs) are emerging as key regulators governing fundamental biological processes, and their disorder expression involves in tumorigenesis. SPRY4-IT1 (*SPRY4* intronic transcript 1), a lncRNA derived from an intron within *SPRY4* gene, involves in multiple cancers development. However, the expression pattern and biological function of SPRY4-IT1 in gastric cancer is still not well documented. Hence, we carried out the present study to investigate the potential role of SPRY4-IT1 in gastric carcinogenesis.

**Methods:**

QRT-PCR was performed to detect the expression of SPRY4-IT1 in 61 pairs of gastric cancer samples. Over-expression and RNA interference (RNAi) approaches were used to investigate the biological functions of SPRY4-IT1. The effect of SPRY4-IT1 on proliferation was evaluated by MTT and colony formation assays. Gastric cancer cells transfected with pCDNA-SPRY4-IT1 were injected into nude mice to study the effect of SPRY4-IT1 on tumorigenesis and metastasis in vivo. Protein levels of SPRY4-IT1 targets were determined by western blot or fluorescence immunohistochemistry. ChIP assays were performed to investigate the effect of DNMT1 on SPRY4-IT1 expression. Differences between groups were tested for significance using Student’s t test (two-tailed).

**Results:**

SPRY4-IT1 expression is decreased in gastric cancer tissues and associated with larger tumor size, advanced pathological stage, deeper depth of invasion and lymphatic metastasis. Patients with lower SPRY4-IT1 expression had a relatively poor prognosis. DNA methylation may be a key factor in controlling the SPRY4-IT1 expression. Furthermore, SPRY4-IT1 contributed to gastric cancer cells metastasis might partly via regulating epithelial–mesenchymal transition (EMT) process.

**Conclusion:**

Low expression of SPRY4-IT1 is involved in progression and metastasis of gastric cancer and may represent a novel biomarker of poor prognosis in patients with gastric cancer.

**Electronic supplementary material:**

The online version of this article (doi:10.1186/s12967-015-0595-9) contains supplementary material, which is available to authorized users.

## Background

Gastric cancer is the second leading cause of cancer related death worldwide, and gastric cancer remains one of the most common malignancies, especially in East Asia [1]. In most patients, gastric cancer is diagnosed at an advanced stage and is accompanied by malignant proliferation, extensive invasion and lymphatic metastasis. Although the clinical outcome of gastric cancer has gradually improved, the prognosis of patients with advanced disease is still disappointing and the 5-year survival rate of patients with gastric cancer still remains relatively low [2]. Recently, multiple lines of evidence have revealed the contribution of long non-coding RNAs (lncRNAs) in tumorigenesis [3]. Therefore, it is important to identify gastric cancer related lncRNAs and investigate their roles in gastric carcinogenesis.

In the past decade, fast development of sequencing technique and completion of ENCODE (encyclopedia of DNA elements) project have led to the discovery of a new group of RNAs, known as lncRNAs. lncRNAs are more than 200 nt in length with limited or no protein-coding capacity, which is often expressed in a disease-, tissue- or developmental stage-specific manner [4–6]. Although lncRNAs are less well characterized compared with microRNAs, increasing evidence suggest that lncRNAs could play critical roles in regulation of diverse cellular processes such as stem cell pluripotency, cell differentiation, cell growth, cell apoptosis and cancer metastasis [7–13]. Additionally, lncRNAs may function as *cis*- or *trans*-regulators of gene expression via playing as scaffolds for chromatin modifying complexes, decoys to transcription factors or serving as ‘sponge’ to microRNAs [14–17].

SPRY4-IT1 (*SPRY4* intronic transcript 1), a lncRNA derived from an intron within *SPRY4* gene, has been recently revealed as oncogenic regulatory hubs or tumor suppressors in different cancers. SPRY4-IT1 was firstly reported to be over-expressed in melanoma cells, and knockdown of its expression inhibited cell growth, invasion and induced cell apoptosis [11, 18]. Moreover, elevated expression of SPRY4-IT1 was associated with poor prognosis of clear cell renal cell carcinoma and esophageal squamous cell carcinoma [19, 20]. SPRY4-IT1 also involved in trophoblast cells proliferation, migration and apoptosis [21]. In previous study, we found that SPRY4-IT1 is down-regulated in non small cell lung cancer, and SPRY4-IT1 could function as a tumor suppressor via regulating cell growth and invasion [22]. However, the expression pattern and biological roles of SPRY4-IT1 in gastric cancer is not well documented. The purpose of this study is to investigate the expression pattern and clinical significance of SPRY4-IT1 in gastric cancer, and identify its key role in gastric cancer cell proliferation and metastasis. This study may advance our understanding of the role of SPRY4-IT1 as a regulator of pathogenesis of gastric cancer and facilitate the development of lncRNA-directed diagnostics and therapeutics.

## Methods

### Tissue collection

61 Paired gastric cancer tissues and normal tissues were obtained from patients who had underwent surgery at Jiangsu province hospital between 2009 and 2011, and were diagnosed with gastric cancer (stages I, II, III, and IV; seventh edition of the *AJCC Cancer Staging Manual*) based on histopathological evaluation. No local or systemic treatment was conducted in these patients before the operation. All specimens were immediately frozen in liquid nitrogen, and stored at −80 °C until RNA extraction. This study was approved by the Research Ethics Committee of Nanjing Medical University, China. Informed consents were obtained from all patients.

### Cell lines and culture conditions

Six gastric cancer cell lines (SGC7901, BGC823, MGC803, AGS, MKN45, MKN28, HCG-27), and a normal gastric epithelium cell line (GES-1) were purchased from the Institute of Biochemistry and Cell Biology of the Chinese Academy of Sciences (Shanghai, China). Cells were cultured in RPMI 1640 or DMEM (GIBCO-BRL) medium supplemented with 10% fetal bovine serum (10% FBS), 100 U/mL penicillin, and 100 mg/mL streptomycin in humidified air at 37°C with 5% CO_2_.

### RNA extraction and qRT-PCR analysis

Total RNA was extracted from tissues or cultured cells using TRIZOL reagent (Invitrogen, Carlsbad, CA). For qRT-PCR, 1 µg RNA was reverse transcribed to cDNA by using a Reverse Transcription Kit (Takara, Dalian, China). Real-time PCR analyses were performed with SYBR Premix ExTaq II kit (Takara, Dalian China). Results were normalized to the expression of GAPDH. The PCR primers were shown in Additional file 1: Table S1. The qRT-PCR assays and data collection were performed on ABI 7500, and results were analyzed and expressed relative to threshold cycle values (ΔCt), then converted to fold changes using the 2^−ΔΔCt^ method. GAPDH was used as an internal control.

### Treatment cells with 5-aza-CdR

BGC823 and SGC7901 cells (2.5 × 10^5^) were seeded into six-well culture plate on day 0 and exposed to 0, 5 µM 5-aza-CdR(Sigma-Aldrich, USA)for 3 days. The cells treated with 5-aza-CdR were harvested and used for detection of SPRY4-IT1 expression.

### Chromatin immunoprecipitation assays

The ChIP assays were performed using EZ-Magna CHIP KIT according to the manufacturer’s instruction (Millipore, Billerica, MA, USA). SGC7901 and BGC823 cells were treated with formaldehyde and incubated for 10 min to generate DNA–protein cross-links. Cell lysates were then sonicated to generate chromatin fragments of 200–300 bp and immunoprecipitated with DNMT1 (Millipore) or the negative control IgG (Millipore). Anti-AcH3 (Millipore) was used as the positive control for the CHIP procedure. Precipitated chromatin DNA was recovered and analyzed by qRT-PCR (Additional file 1: Table S1).

### Transfection of gastric cancer cells

SPRY4-IT1 over-expression plasmid and siRNA has been described in previous study [22]. siRNAs for the human DNMT1 and the negative control olignucleotides were purchased from Invitrogen (Invitrogen, Carlsbad, CA, USA) (Additional file 1: Table S1). All plasmid vectors for transfection (pCDNA-SPRY4-IT1 and empty vector) were extracted by DNA Midiprep or Midiprep kit (Qiagen, Hilden, Germany). Gastric cells cultured on six-well plate were transfected with the pCDNA-SPRY4-IT1, si-SPRY4-IT1 or si-DNMT1 using Lipofectamine 2000 (Invitrogen, Carlsbad, CA, USA) according to the manufacturer’s instructions. Cells were harvested after 48 h for qRT-PCR and western blot analyses.

### Cell proliferation assays

Cell proliferation was monitored using Cell Proliferation Reagent Kit I (MTT) (Roche Applied Science). Si-SPRY4-IT1-transfected BGC823 cells (3,000/well), and pCDNA-SPRY4-IT1-transfected BGC823 or SGC7901 cells (3,000/well) were allowed to grow in 96-well plates. Cell proliferation was documented every 24 h following the manufacturer’s protocol. All experiments were performed in quadruplicate. For the colony formation assay, a total of 500 cells were placed in a fresh six-well plate and maintained in media containing 10% FBS, replacing the medium every 4 days. After 14 days, cells were fixed with methanol and stained with 0.1% crystal violet (Sigma-Aldrich). Visible colonies were manually counted. For each treatment group wells were assessed in triplicate.

### Cell migration and invasion assays

For the transwell assays, at 48 h post-transfection, 5 × 10^4^ (migration) or 1 × 10^5^ (invasion) cells in serum-free media were placed into the upper chamber of an insert (8-μm pore size; Millipore, Billerica, MA, USA). Medium containing 10% FBS was added to the lower chamber. After incubation for 24 h, the cells remaining on the upper membrane were removed with cotton wool, whereas the cells that had migrated or invaded through the membrane were stained with methanol and 0.1% crystal violet, imaged, and counted using an IX71 inverted microscope (Olympus, Tokyo, Japan). Experiments were independently repeated three times.

### Tumor formation assay in a nude mouse model

5 weeks female athymic BALB/c nude mice were maintained under pathogen-free conditions and manipulated according to protocols approved by the Committee on the Ethics of Animal Experiments of Nanjing medical University. BGC823 cells transfected with pCDNA-SPRY4-IT1 or empty vector were harvested from six-well cell culture plates and resuspended at a concentration of 1 × 10^8^ cells/mL. A volume of 100 µL of suspended cells was subcutaneously injected into a single side of the posterior flank of each mouse. Tumor growth was examined every 3 days, and tumor volumes were calculated using the equation V = 0.5 × D × d^2^ (V, volume; D, longitudinal diameter; d, latitudinal diameter). At 15 days post injection, the mice were euthanized and tumor weights were measured and also used for further analysis.

### Tail vein injection of cells for metastasis in athymic mice

5-weeks-old male athymic mice were purchased from the Animal Center of the Nanjing University (Nanjing, China) and maintained in laminar flow cabinets under specific pathogen-free conditions. BGC823 cells transfected with pCDNA-SPRY4-IT1 or the empty vector were harvested from 6-well plates and resuspended at 2 × 10^7^ cells/mL. A volume of 0.1 mL of suspended cells was injected into the tail veins of mice, which were sacrificed 7 weeks after injection. The lungs were removed and photographed, and visible tumors on the lung surface were counted. This study was carried out in strict accordance with the Guide for the Care and Use of Laboratory Animals of the National Institutes of Health. Our protocol was approved by the Committee on the Ethics of Animal Experiments of Nanjing Medical University. All surgery was performed under sodium pentobarbital anesthesia, and all efforts were made to minimize suffering.

### Western blotting analysis

Cells were lysed with RIPA protein extraction reagent (Beyotime, Beijing, China) supplemented with a protease inhibitor cocktail (Roche, CA, USA). The concentration of protein was determined using the Bio-Rad protein assay kit. Protein extracts (40 µg) were separated by 10% SDS-polyacrylamide gel electrophoresis (SDS-PAGE), then transferred to nitrocellulose membranes (Sigma) and incubated with antibodies. ECL chromogenic substrate was used to visualize the bands and the intensity of the bands was quantified by densitometry (Quantity One software; Bio-Rad, CA, USA). GAPDH was used as a control. Antibodies against E-cadherin and Vimentin (1:1,000 dilution) was purchased from Cell Signaling Technology (CST, MA, USA).

### Fluorescence immunohistochemistry

BGC823 cells were fixed in 4% paraformaldehyde following a standard protocol. Rabbit anti-E-cadherin and Vimentin polyclonal antibodies (1:50; CST) were used as primary antibodies, with TRITC-labeled anti-Rabbit IgG (1:200; Sigma-Aldrich, St. Louis, MO, USA) used as a secondary antibody. Sections were mounted onto slides using Gel Mount Aqueous Mounting Medium (G0918, Sigma-Aldrich, St. Louis, MO, USA) and examined with an Olympus BX51 microscope (Olympus, Tokyo, Japan).

### Statistical analysis

Statistical analysis was performed using the SPASS17.0 statistical software package. The expression levels of SPRY4-IT1 in tumor tissues were compared with normal adjacent mucosa by Wilcoxon test, while the associations between SPRY4-IT1 expression and clinical characteristics were evaluated by Chi square test. Survival curves were estimated by the Kaplan–Meier method. The log-rank test was used to estimate the statistical differences between survival curves. A two-tailed P < 0.05 was considered significantly.

## Results

### Decreased expression of SPRY4-IT1 in human gastric cancer

**Fig. 1:**
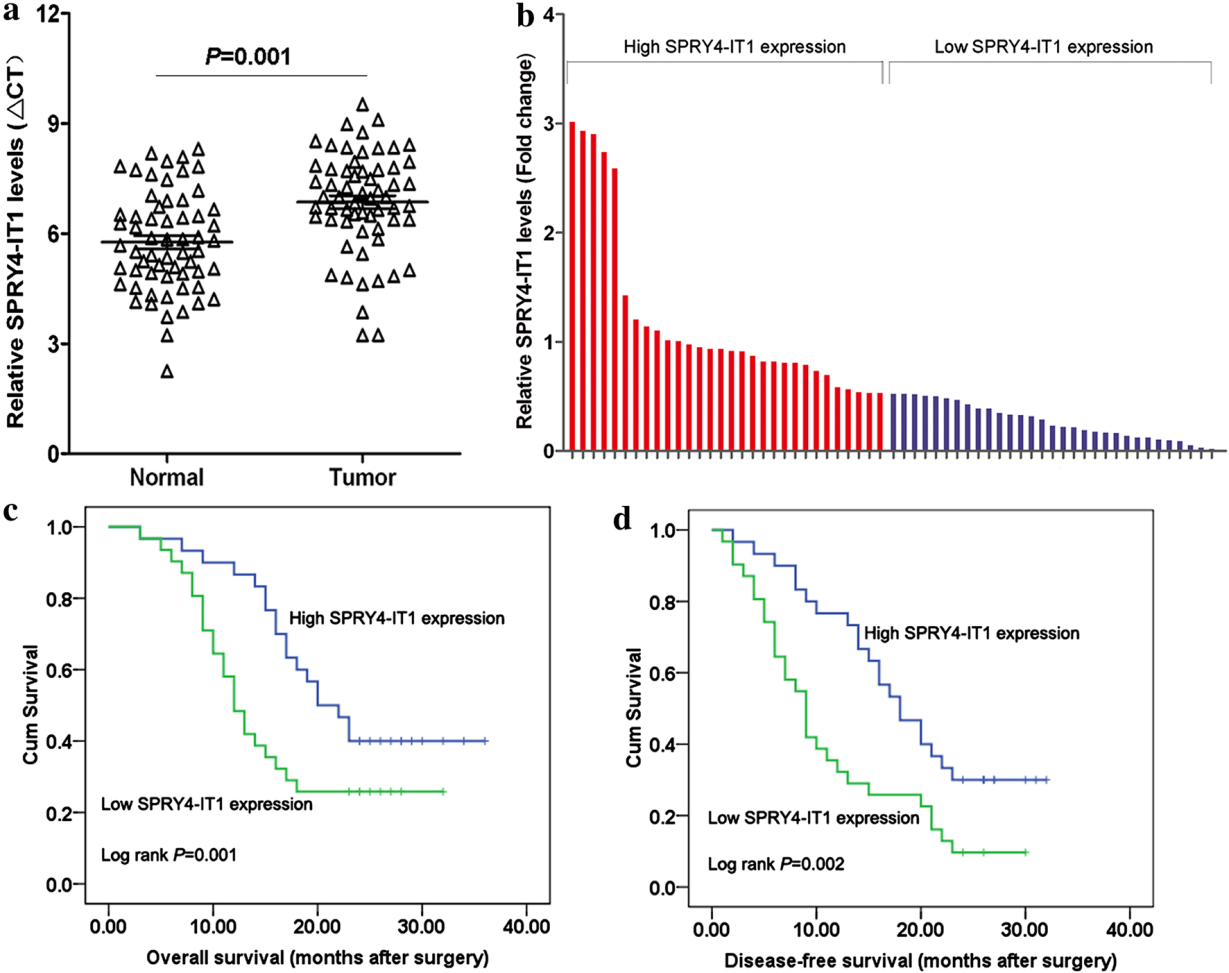
Relative SPRY4-IT1 expression and its clinical significance in gastric cancer tissues. **a** SPRY4-IT1 expression in matched cancerous tissues and adjacent noncancerous tissues from 61 gastric cancer patients were measured by qRT-PCR. Relative gene expression determinations were made with the comparative delta CT normalized to GAPDH expression (*P* = 0.001). **b** According to the median ratio of relative SPRY4-IT1 expression (0.535) in tumor tissues, SPRY4-IT1 expression was classified into two groups: relative high-SPRY4-IT1 group (n = 30, *red column*) and relative low- SPRY4-IT1 group (n = 31, *blue column*). Data was presented as fold-change in tumor tissues relative to normal tissues (*P* = 0.001). **c**, **d** Kaplan–Meier overall survival and disease-free survival curves according to SPRY4-IT1 expression level. Patients with decreased SPRY4-IT1 expression (n = 31) showed reduced survival times compared with patients with high level of SPRY4-IT1 expression (n = 31) (*P* = 0.001 or 0.002, log-rank test).

We firstly examined SPRY4-IT1 expression level in 61 paired gastric cancer samples and adjacent normal tissues using qRT-PCR approach, and gene expression was calculated relative to that of an internal control (ΔCt). As shown in Fig. 1a, the SPRY4-IT1 level was significantly down-regulated in gastric cancer tissue compared with corresponding adjacent non-tumor tissues. (*P* = 0.001, Wilcoxon test). These data indicate that SPRY4-IT1 expression may be related to gastric cancer pathogenesis.

### Correlation between SPRY4-IT1 expression and clinicopathological factors in gastric cancer

Moreover, the correlation between SPRY4-IT1 expression and clinicopathological parameters were examined. According to the median ratio of relative SPRY4-IT1 expression (0.535), the 61 gastric cancer patients were classified into two groups: relative high-SPRY4-IT1 group (n = 30, SPRY4-IT1 expression ratio ≥ median ratio) and relative low-SPRY4-IT1 group (n = 31, SPRY4-IT1 expression ratio ≤ median ratio) (Fig. 1b). Decreased SPRY4-IT1 expression was significantly associated with larger tumor size (*P* = 0.041), advanced pathological stage (*P* = 0.028), deeper depth of invasion (*P* < 0.001) and lymphatic metastasis (*P* = 0.021). However, SPRY4-IT1 expression was not associated with other parameters such as age (*P* = 0.446), gender (*P* = 0.300) tumor location (*P* = 0.931) and histologic differentiation (*P* = 0.155) in gastric cancer (Table 1). Thus, down-regulation of SPRY4-IT1 might have important roles in gastric cancer development and progression.

**Table 1:** Correlation between SPRY4-IT1 expression and clinicopathological characteristics in gastric cancer

Clinical parameter	SPRY4-IT1	SPRY4-IT1	Chi-squared test P value
	High no. cases	Low no. cases	
Age (years)			0.446
<50	13	17	
>50	17	14	
Gender			0.300
Male	16	21	
Female	14	10	
Location			0.931
Distal	11	12	
Middle	12	13	
Proximal	7	6	
Size			0.041*
>5 cm	11	20	
<5 cm	19	11	
Histologic differentiation			0.155
Well	2	2	
Moderately	12	7	
Poorly	14	17	
Undifferentiated	2	5	
Invasion depth			<0.001*
T1	11	3	
T2	12	5	
T3	4	12	
T4	3	11	
TNM stages			0.028*
I	8	1	
II	12	10	
III	9	17	
IV	1	3	
Lymphatic metastasis			0.021*
Yes	11	21	
No	19	10	

### Association between SPRY4-IT1 expression and patients’ survival

We further examined whether SPRY4-IT1 expression level correlated with outcome of gastric cancer patients. Overall survival (OS) and disease-free survival (DFS) curves were plotted according to SPRY4-IT1 expression level by the Kaplan–Meier analysis and log-rank test. Remarkably, patients with low SPRY4-IT1 expression level had poorer disease-free survival (*P* = 0.002) and overall survival (*P* = 0.001) (Fig. 1c, d). The overall 3-year survival rates of patients with high SPRY4-IT1 expression were 40%, while 25.8% for patients with low SPRY4-IT1 expression indicated a shorter overall survival time of patients (median OS: 14 months) compared with high SPRY4-IT1 expression (median OS: 22 months). Moreover, 3 years of disease-free survival for high SPRY4-IT1 expression was 30%, while was 9.7% for low SPRY4-IT1 expression. The median survival time for high SPRY4-IT1 expression is 18 months, while is 9 months for low SPRY4-IT1 expression. These results suggested reduced SPRY4-IT1 expression in gastric cancer may represent a novel indicator of poor prognosis in gastric cancer.

### DNMT1 suppresses SPRY4-IT1 expression in gastric cancer cells

**Fig. 2:**
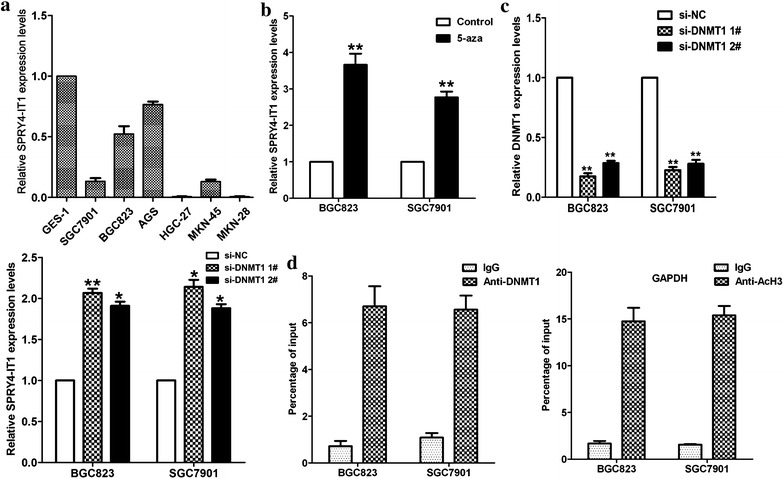
DNMT1 suppresses SPRY4-IT1 expression in gastric cancer cells. **a** QRT-PCR analysis of SPRY4-IT1 expression levels in gastric cancer cell lines (SGC7901, AGS, MKN-45, MKN-28, HGC27 and BGC823) compared with normal human gastric epithelial cell line (GES-1). **b** qRT-PCR analyses of SPRY4-IT1 expression level following treatment of BGC823 and SGC7901 cells with 5-aza-CdR. **c** qRT-PCR analyses of DNMT1 or SPRY4-IT1 expression level following treatment of BGC823 and SGC7901 cells with si-DNMT1. **d** ChIP analyses of DNMT1 binding ability to SPRY4-IT1 promoter region, and anti-AcH3 was used as the positive control binding to GAPDH promoter region. Fold changes using the 2^−ΔΔCt^ method. IgG was used as a negative control. *P < 0.05 and **P < 0.01.

To explore the mechanism of decreased expression of SPRY4-IT1, qRT-PCR was performed to detect the expression of SPRY4-IT1 in a variety of cell lines. The results showed that SPRY4-IT1 expression was significantly down-regulated in gastric cancer cells when compared with GES-1 (Fig. 2a). We hypothesized that epigenetic modification may be involved in aberrant SPRY4-IT1 transcriptional inactivation. Bioinformatic analysis identified a canonical CpG island in the promoter region of the SPRY4-IT1 loci, as described in our previous study [22]. In addition, we found that SPRY4-IT1 expression was significantly increased by 3.7- and 2.8-fold in 5-aza-CdR treated cells (BGC823 and SGC7901) compared with respective controls (Fig. 2b). Moreover, knockdown of DNMT1 expression could also up-regulate SPRY4-IT1 expression in BGC823 and SGC7901 cells (Fig. 2c). To further investigate whether DNMT1 could directly bind to SPRY4-IT1 promoter region, chromatin immunoprecipitation (ChIP) assays were performed on BGC823 or SGC7901 cells using antibodies against DNMT1. As shown in Fig. 2d, enriched DNMT1 immunoprecipitation was observed at the promoter of SPRY4-IT1 in BGC823 and SGC7901 cell lines relative to the IgG immunoprecipitates. Acetylated histone H3 (AcH3) served as a positive control for ChIP assay. Our findings emphasize that DNMT1 mediated DNA methylation may be an factor in controlling SPRY4-IT1 expression.

### Effect of SPRY4-IT1 on gastric cancer cells proliferation in vitro

In order to manipulate SPRY4-IT1 levels in gastric cancer cells, pCDNA-SPRY4-IT1 vector was transfected into SGC7901 and BGC823 cells. QRT-PCR analysis was performed 48 h post-transfection, and revealed that SPRY4-IT1 expression was increased in SGC7901 and BGC823 cells. Furthermore, SPRY4-IT1 siRNAs were transfected into BGC823 cells to down-regulate endogenous SPRY4-IT1 expression, qRT-PCR analysis showed that SPRY4-IT1 expression was effectively knocked down compared with si-NC control cells (Additional file 2: Figure S1A).

**Fig. 3:**
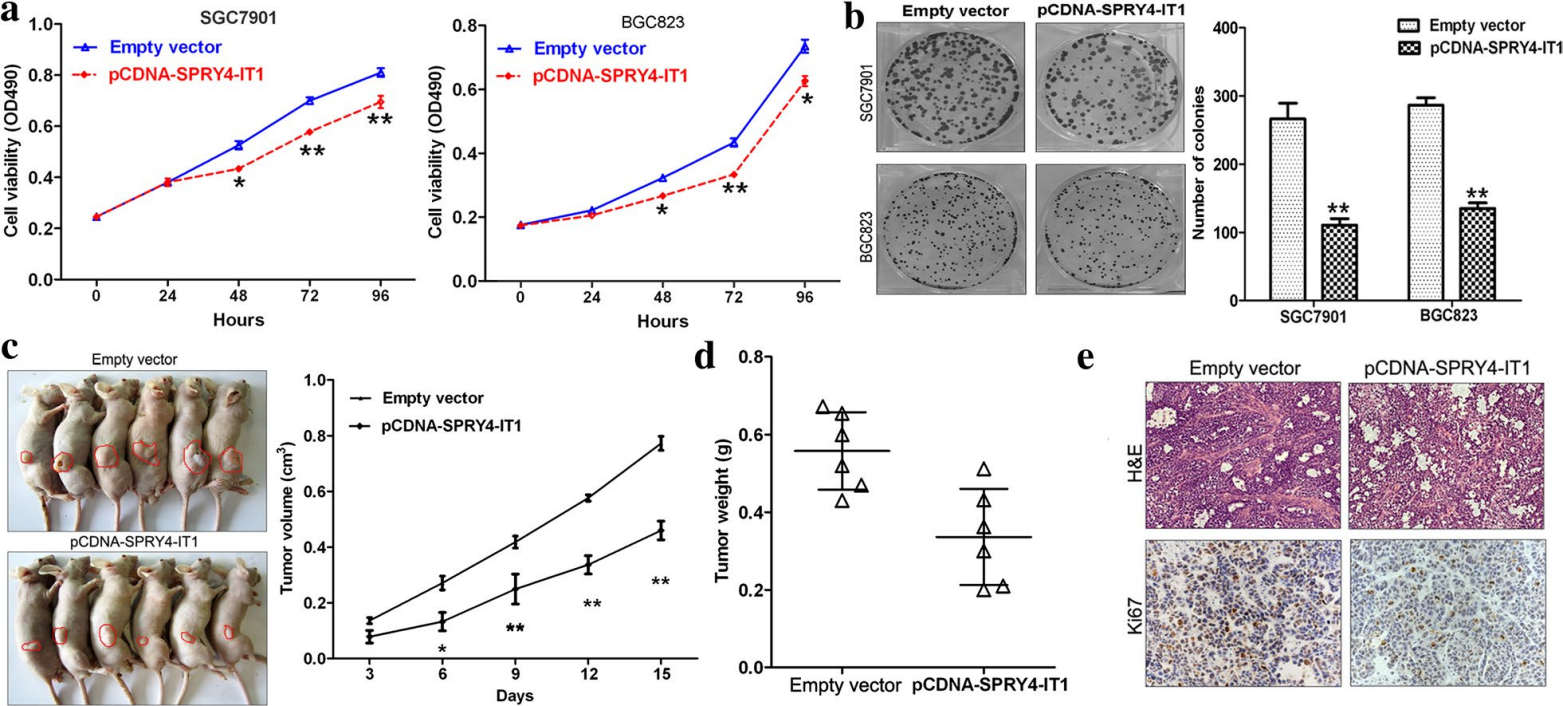
The effect of SPRY4-IT1 on gastric cancer cells proliferation in vitro and vivo. **a** MTT assay was performed to determine the proliferation of pCDNA-SPRY4-IT1 or si-SPRY4-IT1 transfected BGC823 and SGC7901 cells. Data represent the mean ± SD from three independent experiments. **b** Colony-forming growth assay was performed to determine the colony formation ability of pCDNA-SPRY4-IT1 transfected BGC823 and SGC7901 cells. The colonies were counted and captured. **c** The tumor volume was calculated every 3 days after injection of BGC823 cells stably transfected with pCDNA-SPRY4-IT1 or empty vector. **d** Tumor weights are represented as means of tumor weights ± SD. **e** Tumors developed from pCDNA-SPRY4-IT1 transfected BGC823 cells showed lower ki67 protein levels than tumors developed by control cells. *Upper* H & E staining; *lower*: immunostaining. *P < 0.05 and **P < 0.01.

To assess the biological role of SPRY4-IT1 in gastric cancer, we firstly investigated the effect of over-expression of SPRY4-IT1 on cell proliferation. MTT assays showed that cell growth was significantly impaired in pCDNA-SPRY4-IT1 transfected SGC7901 cells or BGC823 cells (Fig. 3a), while knockdown of SPRY4-IT1 expression promoted BGC823 cells proliferation (Additional file 2: Figure S1B). Similarly, the results of colony-formation assays showed that clonogenic survival was decreased following SPRY4-IT1 over-expression in SGC7901 cells or BGC823 cells (Fig. 3b).

### SPRY4-IT1 over-expression inhibits gastric cancer cells tumorigenesis in vivo

To explore whether SPRY4-IT1 could affect tumorigenesis, BGC-823 cells transfected with pCDNA-SPRY4-IT1 or empty vector were used in a nude mice xenograft model. Fifteen days after injection, the tumors formed in pCDNA-SPRY4-IT1 group were substantially smaller than those in the control group (Fig. 3c). Moreover, the mean tumor weight at the end of the experiment was markedly lower in the pCDNA-SPRY4-IT1 group compared to the empty vector group (Fig. 3d). In addition, the SPRY4-IT1 expression levels were up-regulated in tumor tissues collected from pCDNA-SPRY4-IT1 group when compared with control group (Additional file 2: Figure S1C). Next, immunostaining analysis of the Ki67 was performed in resected tumor tissues. In comparison with that in tumors formed from control cells, pCDNA-SPRY4-IT1 derived tumors showed significantly reduced Ki67 positivity (Fig. 3e).

### Effect of SPRY4-IT1 on gastric cancer cells migration and invasion

**Fig. 4:**
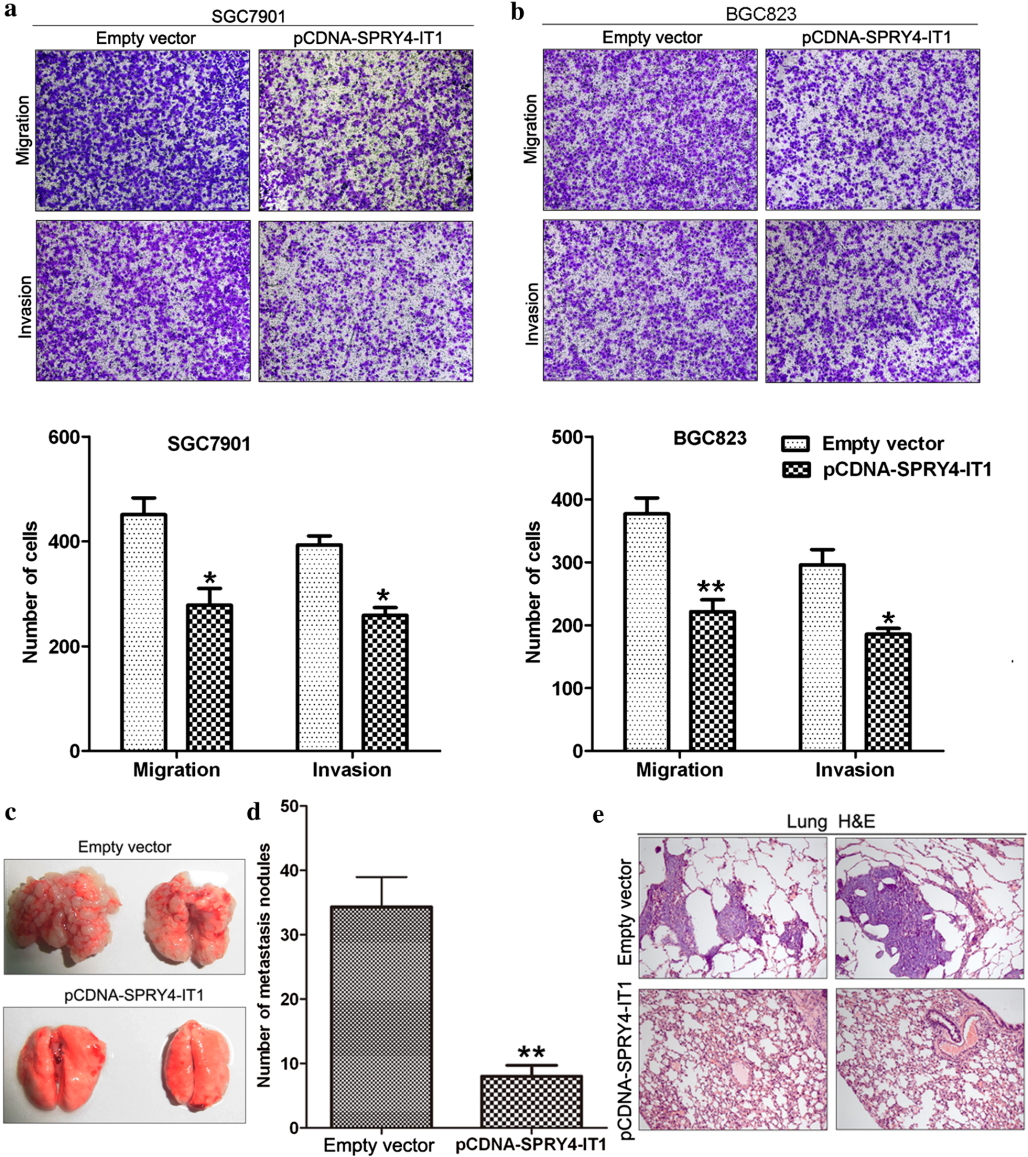
The effect of SPRY4-IT1 on gastric cancer cells invasion and metastasis. **a**, **b** Transwell assays were used to investigate the changes in migratory and invasive abilities of BGC823 and SGC7901 cells transfected with pCDNA-SPRY4-IT1 or empty vector. **c**, **d** Analysis of an experimental metastasis was performed by injecting SPRY4-IT1-overexpressing BGC823 cells into nude mice. **e** Visualization of the entire lungs and HE-stained lung sections. *P < 0.05 and **P < 0.01.

Cell invasion is a significant aspect of cancer progression, and involves the migration of tumor cells into contiguous tissues and the dissolution of extracellular matrix proteins. To investigate whether SPRY4-IT1 has a direct functional role in facilitating gastric cancer cell migration and invasion, we evaluated cancer cell migration and invasion through transwell assays. Increased SPRY4-IT1 expression levels impeded the migration of SGC7901 and BGC823 cells compared with controls. Similarly, invasion of SGC7901 and BGC823 cells was also reduced following up-regulation of SPRY4-IT1 expression (Fig. 4a, b). Conversely, knockdown of SPRY4-IT1 expression enhanced gastric cancer cells migration and invasion ability (Additional file 2: Figure 1D).

### Increased SPRY4-IT1 suppresses gastric cancer cell metastasis in vivo

To validate the effects of SPRY4-IT1 on the metastasis of gastric cancer cells in vivo, BGC823 cells stably transfected with pCDNA-SPRY4-IT1 were injected into nude mice via the tail vein. Metastatic nodules on the surface of the lungs were counted after 7 weeks. Ectopic over-expression SPRY4-IT1 resulted in a reduction of the number of metastatic nodules compared with those in the control group (Fig. 4c, d). This difference was further confirmed following examination of the entire lungs, and through hematoxylin and eosin (HE) staining of lung sections (Fig. 4e). Our in vivo data complemented the results of functional in vitro studies involving SPRY4-IT1.

### SPRY4-IT1 influences gastric cancer cell epithelial–mesenchymal transition

**Fig. 5:**
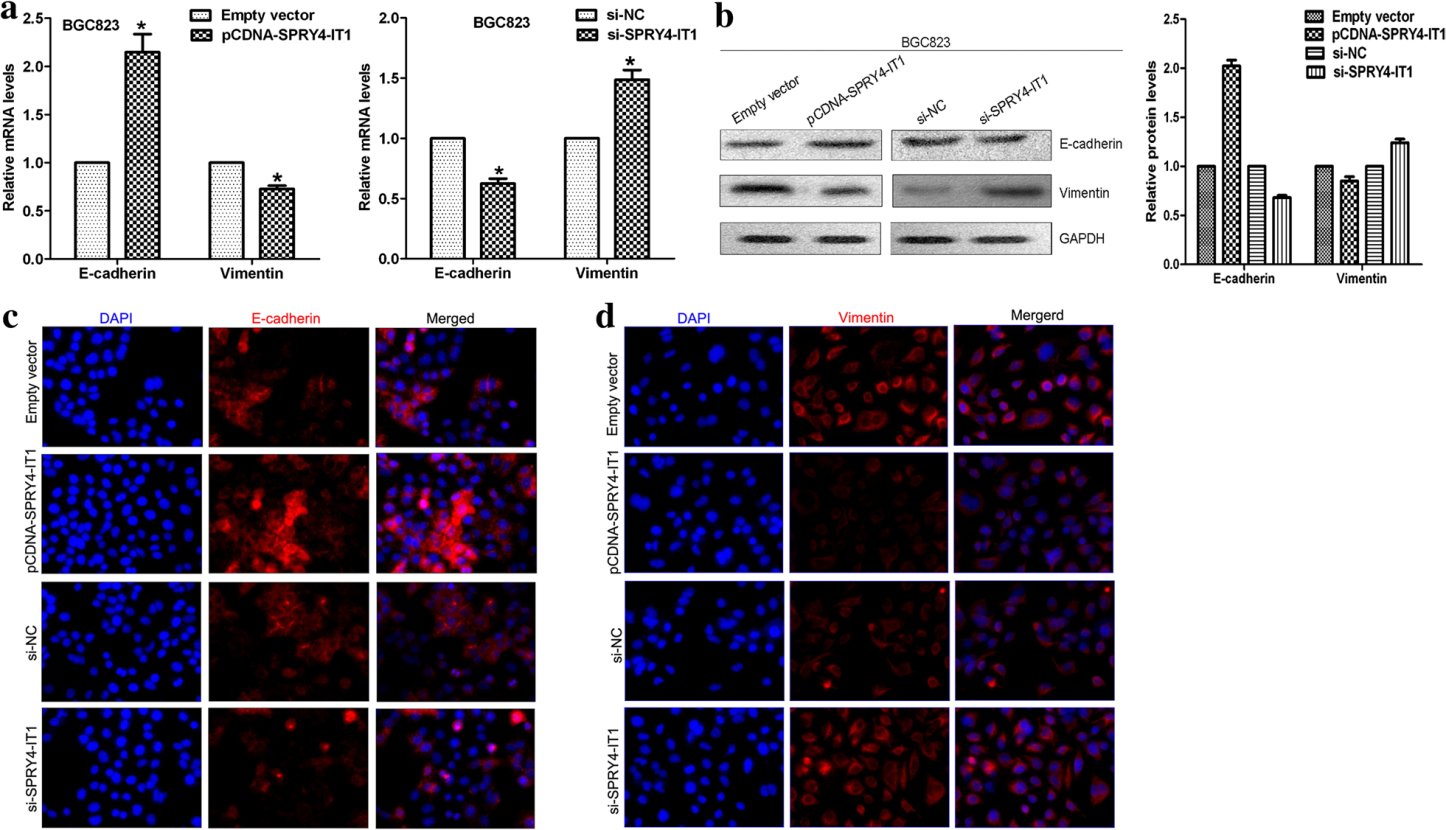
SPRY4-IT1 influences gastric cancer cells epithelial–mesenchymal transition. **a** qRT-PCR assays were performed to detect E-cadherin and Vimentin expression in BGC823 cells transfected with pCDNA-SPRY4-IT1 or si-SPRY4-IT1. **b** Western blot analysis of E-cadherin and Vimentin protein levels in BGC823 cells transfected with pCDNA-SPRY4-IT1 or si-SPRY4-IT1. GAPDH protein was used as an internal control. **c**, **d** Fluorescence immunohistochemistry analysis of E-cadherin and Vimentin protein levels in BGC823 cells transfected with pCDNA-SPRY4-IT1 or si-SPRY4-IT1. *P < 0.05.

As EMT process playing a key role in cancer cells invasion and metastasis, and our previous study indicated that lncRNAs also involved in cancer cells invasion via regulating EMT [23]. In the present study, we determine the expression of the EMT-induced markers in SPRY4-IT1 over-expressed or down-regulated gastric cancer cells. The qRT-PCR results showed that over-expression of SPRY4-IT1 could increase E-cadherin and decrease Vimentin expression, while knockdown of SPRY4-IT1 expression down-regulated E-cadherin expression and up-regulated Vimentin expression (Fig. 5a). In addition, western blot and Fluorescence immunohistochemistry assays showed the same results (Fig. 5b, c and d). These data suggested that SPRY4-IT1 contributes to gastric cancer cells metastasis may partly via affecting EMT process, and further experiments are needed to elucidate the potential mechanism.

## Discussion

Over the past decade, miRNAs have moved to the forefront of ncRNA research in gastric cancer. However, lncRNAs in gastric cancer are still an emerging field. GAPLINC (gastric adenocarcinoma predictive long intergenic noncoding RNA), a 924-bp lncRNA, is highly expressed and displays considerable predictive effect in the diagnosis and prognosis of gastric cancer. Manipulating GAPLINC expression altered CD44 mRNA abundance and regulated cell migration and proliferation by suppressing CD44 expression as a molecular decoy for miR211-3p [24]. Similarly, HOTAIR may function as an completing endogenous RNA to regulate the expression of human epithelial growth factor receptor 2 (HER2) through sponging miR-331-3p [25]. FENDRR is down-regulated in gastric cancer, and decreased expression of FENDRR induces FN1 expression, which resulted in activation of MMP2/MMP9 [26]. Thus, identification of dysregulated lncRNAs will enhance our knowledge of lncRNAs function in the progression and metastasis of gastric cancer and could be used as new diagnostic or therapeutic targets.

SPRY4-IT1 expression was significantly decreased in gastric cancer tissues and cell lines. In addition, the decreased expression of SPRY4-IT1 was associated with poor prognosis and shorter survival time. We also showed that DNMT1 could directly bind to SPRY4-IT1 promoter region and may contribute to the lost expression of SPRY4-IT1 in gastric cancer cells. Moreover, ectopic expression of SPRY4-IT1 led to the significant inhibited malignant phenotype of gastric cancer cells both in vitro and *vivo*. However, SPRY4-IT1 has been found to be up-regulated in esophageal squamous cell carcinoma, breast cancer and bladder cancer, which suggests that SPRY4-IT1 has an tissue-specific expression pattern and may function as oncogene or tumor suppressor in different cancer [20, 27, 28]. Taken together, these findings indicate that SPRY4-IT1 could function as a tumor suppressor and may be useful as a novel prognostic or progression marker for gastric cancer.

The invasion and metastasis of cancer cells are landmark events that involve many changes in cellular behavior, and lead to different steps of the metastatic cascade. Although SPRY4-IT1 can suppress migratory and invasive phenotype of gastric cancer cells, the underlying mechanism is still elusive. We previously showed that SPRY4-IT1 is decreased in NSCLC, and elevation of its expression impairs cell invasion and metastasis through the regulation of EMT process. EMT is a key step toward cancer metastasis, a biological process where epithelial cells lose their polarity and undergo transition into a mesenchymal phenotype. Loss of E-cadherin expression is a hallmark of EMT process and is likely required for enhanced tumor cell motility [29–31]. Perl et al. reported that loss of E-cadherin expression coincides with the transition from well differentiated adenoma to invasive carcinoma in a transgenic mouse model of pancreatic beta-cell carcinogenesis [32]. In this study, we determined the protein levels of these EMT-induced markers following SPRY4-IT1 over-expression or knockdown. Our results indicate that inhibitory effects of SPRY4-IT1 on cell migration and invasion were partly associated with EMT process.

## Conclusions

In summary, we demonstrated that the decreased SPRY4-IT1 expression is a common event underlying the progression and metastasis of gastric cancer, indicating that SPRY4-IT1 may be an indicator of poor survival rate and a negative prognostic factor for gastric cancer patients. SPRY4-IT1 contributed to gastric cancer cells invasion and metastasis may partly via regulating epithelial–mesenchymal transition process. However, the underlying molecular mechanisms through which SPRY4-IT1 involved in EMT requires further investigation.

## Additional files

Additional file 1:
**Table S1.** The primer and siRNA sequence. Additional file 2:
**Figure S1.** Effect of knockdown of SPRY4-IT1 on cell proliferation and invasion. (A) qRT-PCR analyses of SPRY4-IT1 expression level following treatment BGC823 and SGC7901 cells with pCDNA-SPRY4-IT1 or si-SPRY4-IT1. (B) MTT assay was performed to determine the proliferation of si-SPRY4-IT1 transfected BGC823 cells. (C) qRT-PCR analysis of SPRY4-IT1 expression in tumor tissues formed from pCDNA-SPRY4-IT1 or empty vector group. (D) Transwell assays were used to investigate the changes in migratory and invasive abilities of BGC823 cells transfected with si-SPRY4-IT1 or si-NC. *P < 0.05, **P < 0.01.
