# Supplementary figures and images for: Decreased long noncoding RNA SPRY4-IT1 contributing to gastric cancer cell metastasis partly via affecting epithelial–mesenchymal transition
Source: J Transl Med. 2015 Aug 4;13:250. doi: 10.1186/s12967-015-0595-9 (PMC4522960; doi:10.1186/s12967-015-0595-9)

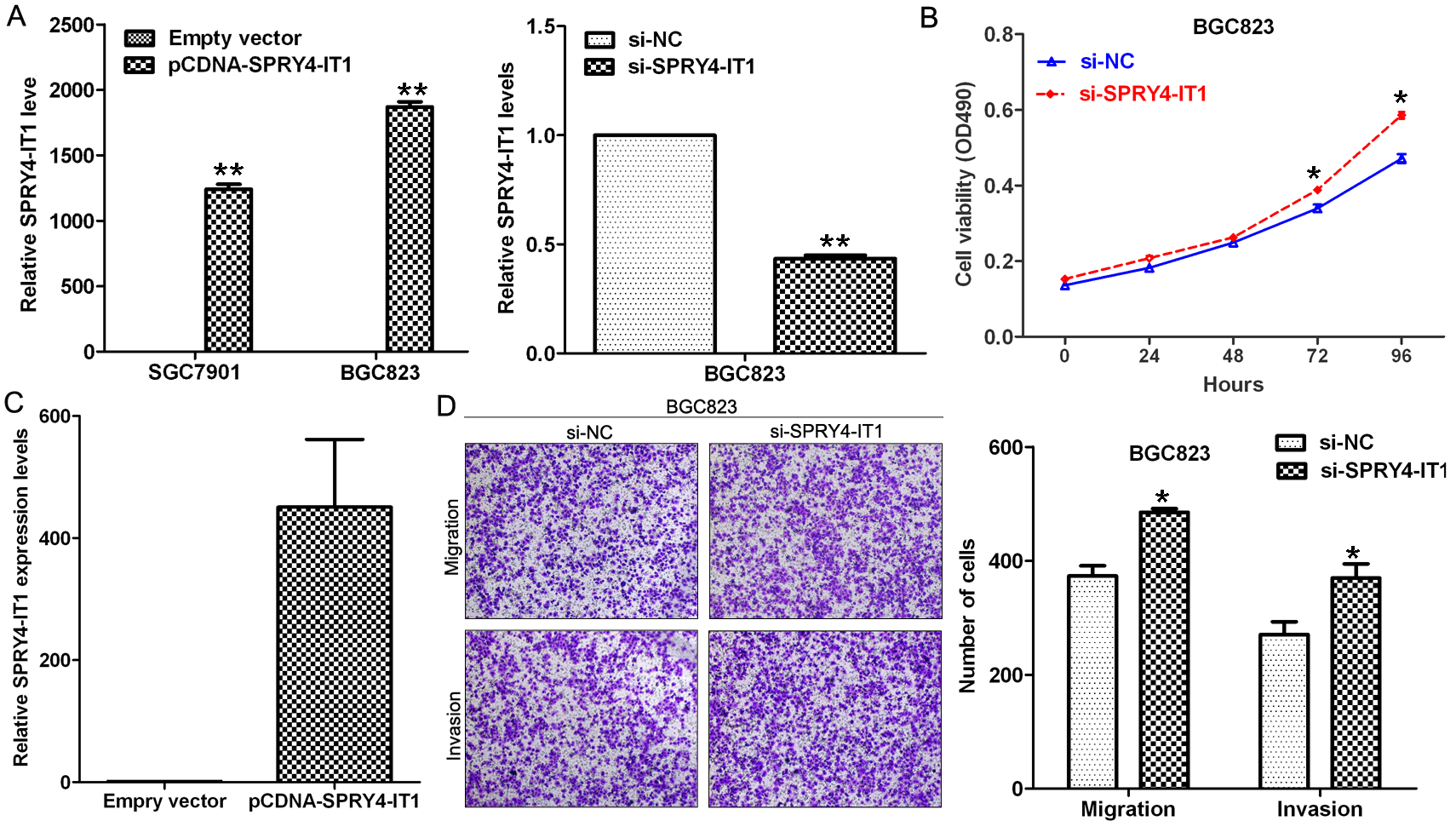

Supplement: Additional file 2: — Figure S1. Effect of knockdown of SPRY4-IT1 on cell proliferation and invasion. (A) qRT-PCR analyses of SPRY4-IT1 expression level following treatment BGC823 and SGC7901 cells with pCDNA-SPRY4-IT1 or si-SPRY4-IT1. (B) MTT assay was performed to determine the proliferation of si-SPRY4-IT1 transfected BGC823 cells. (C) qRT-PCR analysis of SPRY4-IT1 expression in tumor tissues formed from pCDNA-SPRY4-IT1 or empty vector group. (D) Transwell assays were used to investigate the changes in migratory and invasive abilities of BGC823 cells transfected with si-SPRY4-IT1 or si-NC. *P < 0.05, **P < 0.01. [file 12967_2015_595_MOESM2_ESM.tiff]
